# Functional genomics annotation of a statistical epistasis network associated with bladder cancer susceptibility

**DOI:** 10.1186/1756-0381-7-5

**Published:** 2014-04-11

**Authors:** Ting Hu, Qinxin Pan, Angeline S Andrew, Jillian M Langer, Michael D Cole, Craig R Tomlinson, Margaret R Karagas, Jason H Moore

**Affiliations:** 1 Department of Genetics, Geisel School of Medicine, Dartmouth College, Hanover, NH 03755, USA; 2 Institute for Quantitative Biomedical Sciences, Dartmouth College, Hanover, NH 03755, USA; 3 Department of Community and Family Medicine, Geisel School of Medicine, Dartmouth College, Hanover, NH 03755, USA; 4 Department of Pharmacology & Toxicology, Geisel School of Medicine, Dartmouth College, Hanover, NH 03755, USA

**Keywords:** Epistasis, Gene-gene interactions, Statistical epistasis networks, Benzo[*a*]pyrene, Gene-drug association, Bladder cancer

## Abstract

**Background:**

Several different genetic and environmental factors have been identified as independent risk factors for bladder cancer in population-based studies. Recent studies have turned to understanding the role of gene-gene and gene-environment interactions in determining risk. We previously developed the bioinformatics framework of statistical epistasis networks (SEN) to characterize the global structure of interacting genetic factors associated with a particular disease or clinical outcome. By applying SEN to a population-based study of bladder cancer among Caucasians in New Hampshire, we were able to identify a set of connected genetic factors with strong and significant interaction effects on bladder cancer susceptibility.

**Findings:**

To support our statistical findings using networks, in the present study, we performed pathway enrichment analyses on the set of genes identified using SEN, and found that they are associated with the carcinogen benzo[*a*]pyrene, a component of tobacco smoke. We further carried out an mRNA expression microarray experiment to validate statistical genetic interactions, and to determine if the set of genes identified in the SEN were differentially expressed in a normal bladder cell line and a bladder cancer cell line in the presence or absence of benzo[*a*]pyrene. Significant nonrandom sets of genes from the SEN were found to be differentially expressed in response to benzo[*a*]pyrene in both the normal bladder cells and the bladder cancer cells. In addition, the patterns of gene expression were significantly different between these two cell types.

**Conclusions:**

The enrichment analyses and the gene expression microarray results support the idea that SEN analysis of bladder in population-based studies is able to identify biologically meaningful statistical patterns. These results bring us a step closer to a systems genetic approach to understanding cancer susceptibility that integrates population and laboratory-based studies.

## Findings

### Introduction

Tobacco consumption has been implicated as the most relevant risk factor for the development of bladder cancer. Among various carcinogens identified in tobacco smoke, the polycyclic aromatic hydrocarbons (PAHs) are well characterized and their risk associations with bladder cancer are well studied [1-4]. In addition to these strong environmental risk factors, recent genome-wide association studies have identified several highly significant genetic factors with small effects [5,6]. Some of these have been shown to modify the effects of smoking on risk of bladder cancer [7].

Most existing genetic association studies have focused on the independent effects of individual genes. That is, they have by design ignored the context of human ecology and the extensive variability in the human genome. As a result, much of the heritability of common human diseases such as bladder cancer remains unexplained. Multiple approaches have been proposed to account for this missing heritability including sequencing to identify causative rare variants and advanced analytical strategies to characterize those genetic effects that are context-dependent [8]. The characterization of gene-gene interaction effects, i.e. *epistasis*, provides a promising research avenue to understand the complex genetic architecture underlying common human diseases in population-based studies [9-11]. Previously, we developed a network-based framework, statistical epistasis networks (SEN), to characterize the global epistatic interaction space of bladder cancer [12,13]. SEN were able to identify a large set of interacting single-nucleotide polymorphisms (SNPs) that had strong associations with bladder cancer. In addition, these SNPs were organized in the form of a connected network structure that was highly significant and nonrandom compared to the null distribution of networks generated using permutation tests.

The goal of the present study was to determine whether the SEN identified for bladder cancer have any biological relevance in the context of the most prevalent risk factor. Because the majority of bladder cancer cases in the United States can be attributed to tobacco exposure, we hypothesized that many of the SEN genes would have a biological role related to biological response to PAH exposure. Here, we applied pathway enrichment analysis of the bladder cancer SEN and found that it is enriched for genes that have strong associations with benzo[*a*]pyrene. We carried out a gene expression microarray experiment in cell lines from normal bladder and bladder cancer cells treated with and without benzo[*a*]pyrene. The goal of this experiment was to provide a functional genomics annotation of the SEN.

### Methods and results

In the framework of SEN [12], first we exhaustively measured the non-additive statistical interactions among all pairs of SNPs in a large population-based bladder cancer case–control dataset [14]. The information theoretic measurement *information gain* was used to quantify the epistatic interaction between two SNPs associated with bladder cancer [15-17]. The information gain measure was able to separate the pure synergistic interaction effect from the additive main effects of a pair of genetic attributes, and thus to characterize their statistical epistasis associated with a particular disease status or phenotype. Network properties, including network size, global network connectivity, and degree distribution, were analyzed to systematically derive a pairwise interaction strength threshold to construct an interaction network that had significantly distinct structures compared to null networks built from permutation testing [12]. This network had a sizable largest connected component including 39 SNPs from 29 cancer susceptibility genes (Table 1), which were absent in any of the 1000 null networks using permuted data. We speculated that these identified 29 genes captured an important aspect of the underlying complex genetic structure of bladder cancer [12].

**Table 1:** The 29 genes identified in our previous genetic interaction study on bladder cancer using SEN

**Symbol**	**Entrez Gene ID**	**Description**
AHRR	57491	Aryl-hydrocarbon receptor repressor
AKR1C3	8644	Aldo-keto reductase family 1, member C3
AXIN2	8313	Axin2
BCL6	604	B-cell CLL/lymphoma 6
BIRC3	330	Baculoviral IAP repeat containing 3
CARD15	64127	Nucleotide-binding oligomerization domain containing 2
CAT	847	Catalase
CCL5	6352	Chemokine (C-C motif) ligand 5
CCNH	902	Cyclin H
FTHFD	10840	Aldehyde dehydrogenase 1 family, member L1
GATA3	2625	GATA binding protein 3
GSTM3	2947	Glutathione S-transferase mu 3 (brain)
HSD17B4	3295	Hydroxysteroid (17-beta) dehydrogenase 4
IGF1R	3480	Insulin-like growth factor 1 receptor
IL1A	3552	Interleukin 1, alpha
INSR	3643	Insulin receptor
MASP1	5648	Mannan-binding lectin serine peptidase 1 (C4/C2 activating component of Ra-reactive factor)
MBD2	8932	Methyl-CpG binding domain protein 2
MMP1	4312	Matrix metallopeptidase 1 (interstitial collagenase)
MYBL2	4605	V-myb myeloblastosis viral oncogene homolog (avian)-like 2
NEDD5	4735	Septin 2
OPRM1	4988	Opioid receptor, mu 1
PARP4	143	Poly (ADP-ribose) polymerase family, member 4
PGR	5241	Progesterone receptor
PIM1	5292	Pim-1 oncogene
RERG	85004	RAS-like, estrogen-regulated, growth inhibitor
TNKS	8658	Tankyrase, TRF1-interacting ankyrin-related ADP-ribose polymerase
TP53I3	9540	Tumor protein p53 inducible protein 3
XPC	7508	Xeroderma pigmentosum, complementation group C

**Figure 1:**
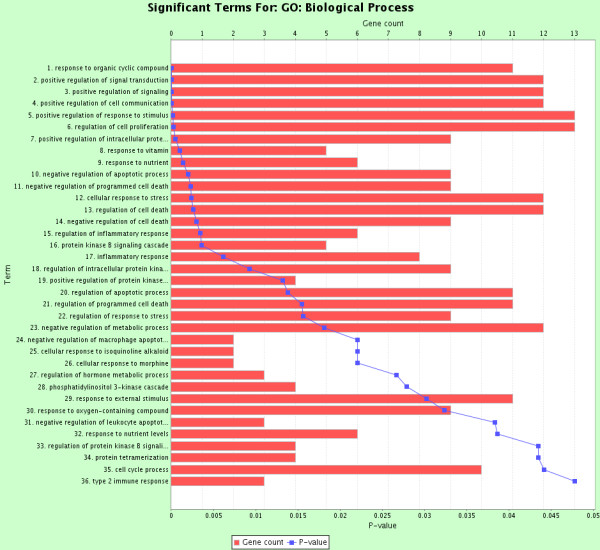
**ToppGene enrichment analysis on the GO biological process.** Terms were considered significant when their significance p < 0.05 (after Bonferroni multiple-testing correction). Bars show the gene counts from our list of 29 genes, and lines show the enrichment significance levels. Terms (y-axis) are ranked based on their p-values.

**Figure 2:**
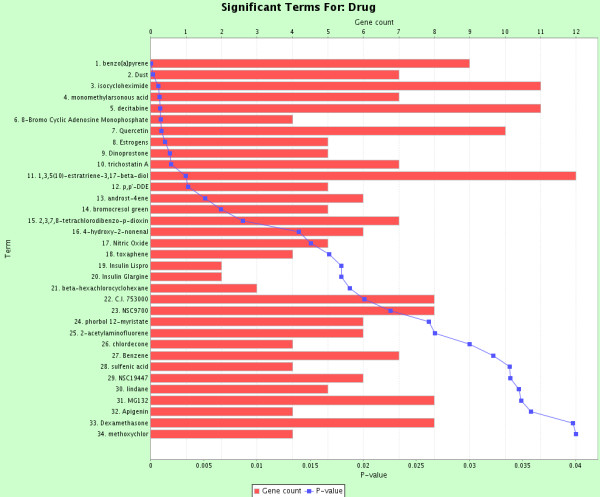
**ToppGene enrichment analysis on the drug-gene association.** Significant terms were chosen with a Bonferroni multiple-testing corrected significance level p < 0.05. Bars show the gene counts from our list of 29 genes, and lines show their enrichment p -values.

To further validate the effectiveness of our population-based SEN methodology at capturing meaningful biology in our bladder cancer epistatic interaction network, we performed functional enrichment analyses on those 29 genes using ToppGene [18]. ToppGene detects functional enrichment of a given gene list based on ontologies, pathways, phenotypes, drug-gene association, etc. All statistical results were corrected for multiple testing using the Bonferroni method. For our list of 29 genes, the most significant biological process from the Gene Ontology analysis was response to organic cyclic compound (p = 1.27*10^-5^). Figure 1 summarizes all 39 significant biological processes. In addition, the most significant chemical from the drug-gene analysis was benzo[*a*]pyrene (p = 2.90*10^-5^). Figure 2 summarizes all 34 significant drug-gene categories. These enrichment results indicate that our SEN successfully identified interacting genes that are mostly targeted and regulated by the carcinogen benzo[*a*]pyrene.

Next, we performed a gene expression microarray experiment by exposing both normal bladder cells and bladder cancer cells to benzo[*a*]pyrene. Four plates each of the normal urothelial cell line UROtsa and the tumorigenic cell line J82 with and without the treatment of 10 uM benzo[*a*]pyrene, were cultured. mRNAs were purified and transcription levels were measured using the HumanHT-12 v3 Expression BeadChip (Illumina, Inc., San Diego, CA). There were 47,230 probes on the array of which 29,785 were kept after filtering for detectable probes (detection p-value ≤ 0.05). A quantile normalization method was applied to normalize the data [19]. To test for genes that were differentially expressed in cell lines with and without benzo[*a*]pyrene treatment, we used a random variance *t*-test as described by Wright et al. [20]. Genes with a parametric p ≤ 0.05 were considered differentially expressed.

**Figure 3:**
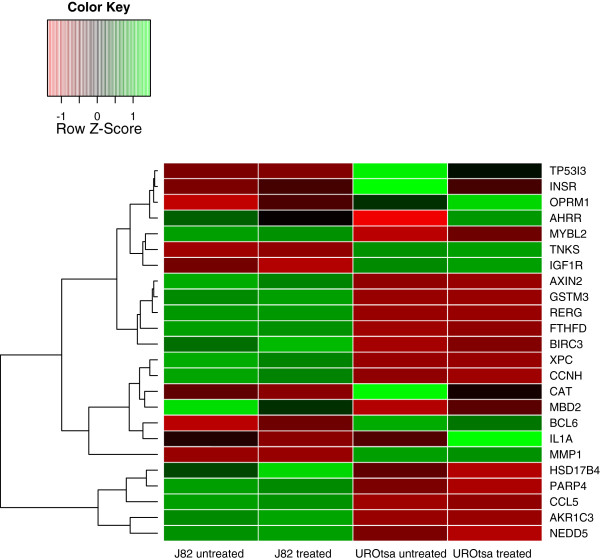
**Gene expression levels in the four groups of samples of our network genes.** The green color indicates up-regulation, and red color indicates down-regulation. Genes are clustered based on their expression patterns across all four groups.

Out of the 29 genes identified in our SEN, 26 had detectable probes on the microarray. Their differential expression patterns are shown in Figure 3. Of these, there were 16 genes that had lower expression levels in the J82 untreated bladder cancer cells compared to the UROtsa untreated cells. When comparing J82 cells with and without the treatment, six genes were identified as differentially expressed. Out of these six genes, *BIRC3*, *AKR1C3*, and *BCL6* were up-regulated with the treatment of benzo[*a*]pyrene, whereas *IL1A*, *MBD2*, and *AXIN2* were down-regulated. For the UROtsa cell line with and without the toxicant treatment, six genes were identified as differentially expressed. Among them, *AHRR* and *IL1A* were up-regulated, whereas *NEDD5*, *INSR*, *CAT*, and *BCL6* were down-regulated with the presence of benzo[*a*]pyrene. It is interesting to note that, although *IL1A* was differentially expressed in both cell lines with and without the toxicant treatment, the direction of the change was opposite.

**Figure 4:**
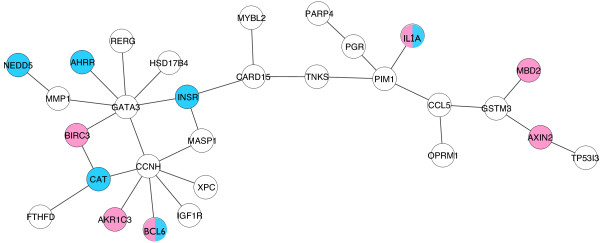
**Gene-gene interaction network annotated with differentially expressed genes in response to benzo[*a*]pyrene.** Each vertex represents a gene and two genes are connected by an edge if they have detected significant and strong statistical epistatic interactions through their underlying gene-coding SNPs. Vertices are labeled with gene symbols and colored with differential expressions in the normal UROtsa cells (blue) and the cancer J82 cells (pink).

We then annotated the genes with significantly differential expressions, either up-regulated or down-regulated, in our SEN (Figure 4). We first transformed the original SNP-SNP interaction network into a gene-gene interaction network by setting each vertex as a gene and connecting two genes if there existed at least one pair of SNPs, each from one of the two genes, that had a significant and strong pairwise interaction in the original SEN. Genes that were differentially expressed in the normal UROtsa cells with and without the toxicant treatment are shown in blue color in Figure 4. Pink colored vertices represent genes that were differentially expressed in the tumorigenic J82 cells with and without the toxicant treatment. Also note that two genes, *IL1A* and *BCL6*, were identified differentially expressed in both the normal and the cancer cells. The *IL1A* gene encodes for interleukin 1 alpha, a member of the interleukin 1 cytokine family. This cytokine is produced by monocytes and macrophages as a proprotein, which is proteolytically processed and released in response to cell injury and thus induces apoptosis. *IL1A* was up-regulated in the normal UROtsa cell but down-regulated in the tumorigenic J82 cells in response to the treatment of benzo[*a*]pyrene. Since a major function of *IL1A* is to induce apoptosis, a mechanism for programmed killing of potentially harmful cells, the identification of *IL1A* both in the SEN and in the differential gene expression experiment suggests its potential role in interacting with the carcinogen and the development of bladder cancer. Likewise, the Aryl-Hydrocarbon Receptor Repressor (*AHRR*) responded differentially to benzo[*a*]pyrene treatment by cell line. Expression levels are increased by tobacco smoke exposure by a mechanism involving less DNA methylation [21]. We observed increased *AHRR* expression in the normal bladder epithelial cells, as expected, however the opposite relationship in the cancer cell line. To provide further validation for our results, we used the ten differentially expressed SEN genes (Figure 4) for another enrichment analysis using ToppGene, and a strong drug association with benzo[*a*]pyrene was detected (p = 3.733*10^-3^).

It is interesting to observe that the normal UROtsa cell and the tumorigenic J82 cells only had two differentially expressed genes in common. The SEN was derived from a population-based case–control study. Genes in the network were those that contained SNPs associated with bladder cancer risk. The fact that their expression levels changed differently in normal and tumorigenic cells supports a biological role for these genes in response to tobacco, that potentially the cells to have greater susceptibility to transformation or carcinogenic progression. The differential responses to benzo[*a*]pyrene in the normal and cancer cells indicate the possible contribution of interactions between genetic factors and the tobacco carcinogen to bladder cancer risk.

### Conclusion

In this study, we used functional genomics data to validate genetic interaction network that was constructed purely based on statistical analysis. We performed an enrichment analysis on a set of genes previously identified by our SEN methodology that had strong and significant epistatic interactions associated with bladder cancer. We tested the hypothesis that these susceptibility genes would be related to tobacco response, since this is the major bladder carcinogen. The enrichment analysis results suggested our network had more genes that respond to a tabacco carcinogen benzo[*a*]pyrene than would be expected by chance. We then carried out a microarray experiment culturing normal bladder and bladder cancer cells in the presence or absence of benzo[*a*]pyrene. These results showed that there were more genes in our SEN that respond to benzo[*a*]pyrene than would be expected by chance given the size of the network. These gene set enrichment and functional genomics annotation analysis results suggest that the SEN methodology may be able to capture biological effects at the heart of the interplay between environmental factors such as smoking and the complex gene-gene interactions that likely influence risk of bladder cancer. Although not functional proof, this study demonstrates how integrating population-based statistical results with cellular experiments can assist with interpretation of associations to provide a systems genetics approach to understanding cancer susceptibility.

## Abbreviations

PAH: Polycyclic aromatic hydrocarbon; SEN: Statistical epistasis networks; SNP: Single-nucleotide polymorphism; GO: Gene Ontology.

## Competing interests

The authors declare no competing interests.

## Authors’ contributions

TH designed the study, performed the analyses, and drafted the manuscript. QP participated in the design of the study, analyzed the microarray experiment results, and participated in drafting the manuscript. ASA and MRK collected the bladder cancer population data and participated in the design of the study. JML, MDC, CRT, and carried out the gene expression experiment and participated in the design of the study. JHM conceived of the study, participated in its design and coordination, and helped drafting the manuscript. All authors read and approved the final manuscript.
